# Impact Evaluation of the Get Healthy in Pregnancy Program: Evidence of Effectiveness

**DOI:** 10.3390/healthcare11172414

**Published:** 2023-08-29

**Authors:** Bronwyn McGill, Dominic Lees, Justine Salisbury, Tahlia Reynolds, Sandy Davidson, Edwina Dorney, Sarah Yeun-Sim Jeong, Blythe J. O’Hara

**Affiliations:** 1 Prevention Research Collaboration, Charles Perkins Centre, Sydney School of Public Health, The University of Sydney, Camperdown, NSW 2006, Australia; 2 Biostatistics Training Program, NSW Ministry of Health, St Leonards, NSW 2065, Australia; 3 Centre for Population Health, NSW Ministry of Health, St Leonards, NSW 2065, Australia; 4 Charles Perkins Centre, Sydney School of Nursing, The University of Sydney, Camperdown, NSW 2006, Australia

**Keywords:** pregnancy, gestational weight gain, prevention, lifestyle intervention, physical activity, healthy eating

## Abstract

The efficacy of lifestyle interventions for reduced gestational weight gain (GWG) is established, but evidence of their effectiveness is limited. The Get Healthy in Pregnancy (GHiP) program is a telephone health coaching program supporting healthy GWG delivered state-wide in New South Wales, Australia. This evaluation explores the impact of GHiP on behavioural outcomes and GWG, analysing GHiP participant data (n = 3702 for 2018–2019). We conducted McNamar’s tests to explore within-individual change for behavioural outcomes and logistic regression to assess associations between demographic characteristics, participant engagement and behavioural and weight outcomes for women who completed the program. Participants who completed ten coaching calls made significant improvements (all *p* < 0.001) in more health-related behaviours (walking, vigorous physical activity, vegetable consumption, takeaway meals and sweetened drink consumption) than those who completed fewer calls. Among women with valid weight change data (n = 245), 31% gained weight below, 33% gained weight within, and 36% gained weight above GWG guidelines. Pre-pregnancy BMI was the only factor significantly associated with meeting GWG guidelines. Women with pre-pregnancy overweight and obesity had lower odds than those with a healthy weight of having GWG within the guidelines. The majority of these women did not gain weight above the guidelines. A higher proportion of women with pre-pregnancy obesity gained weight below the guidelines (33.8%) than above the guidelines (28.5%). GHiP has the potential to support all pregnant women, including those with pre-pregnancy obesity, to achieve a healthier pregnancy.

## 1. Introduction

There is growing recognition that maternal preconception obesity and greater than recommended gestational weight gain (GWG) have negative impacts for mothers and their babies [1,2,3,4,5]. Maternal preconception obesity increases the risk of adverse outcomes including early pregnancy loss, congenital foetal malformations and complications later in pregnancy such as gestational diabetes, pre-eclampsia, delivery of large for gestational age infants, premature birth and still birth [6,7,8].

In 2009, the Institute of Medicine (IOM) provided guidelines for the optimal recommended GWG based on pre-conception body mass index (BMI) [9]. Australian data demonstrates that over half of women classified as being overweight (58.8%) and 36.7% of women classified as obese experienced excessive GWG, and among women with healthy pre-pregnancy weight, 32% also experienced excessive GWG [10]. Notwithstanding the need to identify and address the complex interplay between the multiple demographic and socio-cognitive factors associated with GWG [10], research has been undertaken that has tested the efficacy of interventions designed to reduce excessive GWG [5,11,12,13,14,15,16]. A recent meta-analysis that examined the association between antenatal lifestyle interventions with GWG and maternal and neonatal outcomes found that structured diet and physical activity-based interventions during pregnancy were associated with reduced weight gain, and lower risks of adverse outcomes for both mother and baby [16].

The Get Healthy in Pregnancy (GHiP) program is a free telephone coaching program, aimed to encourage healthy eating and active living behaviours, that is available to pregnant women aged 16+ years in New South Wales (NSW), Australia. GHiP (www.gethealthynsw.com.au) is delivered by university-qualified coaches through a program provider. The program was first offered in July 2015 to achieve healthy GWG in line with the IOM guidelines. The program offers free, personalised telephone-based health coaching (up to 10 confidential calls over six months) during pregnancy, which complements antenatal care. Participants are encouraged to set their own goals for weight, physical activity and diet. Participants are referred to the GHiP program by midwives and maternity services, medical professionals, and other health professionals (e.g., diabetes educators, pharmacists), or can self-refer at any time prior to their baby being born. Postpartum, participants may re-enrol for further coaching to support their maintenance or achievement of a healthy post-pregnancy weight, or to receive six months of SMS-based ongoing support.

A pilot randomised control study of the GHiP program found that more women in the health-coaching arm gained weight within the IOM guidelines at 36 weeks gestation (42.9%) compared to the information-only arm (31.9%) [17]. Additionally, women found GHiP to be helpful, and midwives and doctors noted that it facilitated conversations about weight with pregnant women [18]. Following this pilot study and strong positive feedback that a scaled-up program would be well received [17,18], the GHiP program was delivered across the state of NSW, Australia.

While the available literature supports the efficacy of healthy lifestyle behaviour-based interventions under controlled conditions to manage weight gain during pregnancy, there is limited evidence about the effectiveness of these interventions (i.e., how successfully an intervention achieves the predicted impact and outcomes in real-life conditions) when implemented at scale [19]. There is therefore an opportunity to address a gap in translational evidence for the GHiP program effectiveness. The purpose of this evaluation is to investigate the effectiveness of the GHiP program as it is implemented at scale. Specifically, this evaluation reports (a) participant engagement with the program, and (b) the program’s impact on their health behaviour outcomes and GWG.

## 2. Materials and Methods

### 2.1. Study Design and Participants

This evaluation involves a retrospective analysis of routinely collected data from participants in the GHiP program. Data from women who enrolled in the GHiP program from 1 January 2018 to 31 December 2019 were included. Ethics approval for this study was granted by the University of Sydney Human Ethics Committee (2019/710).

### 2.2. Data Collection

All participants provided data to the GHiP program provider as part of their enrolment and ongoing participation in the program. Participants were asked whether they consented for their data to be used for evaluation, and only data from those who agreed were included in this study. Data were collected at baseline (first coaching call), mid-point (call six) and at program completion; defined as either early goal attainment (after four coaching calls) or graduation (10 calls). 

#### 2.2.1. Sociodemographic Measures

Demographic characteristics reported by participants included age, level of education, employment, language spoken at home, Aboriginality and residential postcode, as well as information regarding previous pregnancies. Postcodes were used to define social disadvantage and geographical remoteness using Socio-Economic Indexes for Areas (SEIFA, Index of Relative Socio-Economic Disadvantage—IRSD) [20] and Accessibility-Remoteness Index of Australia Plus (ARIA) [21], respectively.

#### 2.2.2. Participant Engagement and Program Completion

The number of coaching calls received by participants was recorded by the program provider. Program completion (whether an individual finished the program or not) was denoted by a status of ‘graduated’ (completed all allocated calls) or ‘early goal attainment’ (reached their goal any time after four calls prior to completing 10 coaching calls).

#### 2.2.3. Weight-Related Measures

Self-reported height (cm) and pre-pregnancy weight (kg) were included in referrals from health professionals, and also recorded by the service provider. Pre-pregnancy BMI (kg/m^2^) was used to classify participants according to healthy weight (18.5–24.99 kg/m^2^), overweight (25–29.99 kg/m^2^) or obese (≥30 kg/m^2^) [22]. Self-reported weight (kg) was recorded at baseline, mid-point and at program completion. Weight-related outcomes in this study were based on whether women met the IOM guidelines for GWG depending on the mother’s pre-pregnancy BMI [9], using two approaches. The first approach dichotomously categorised women as either having met or not met guidelines based on determining a final valid weight prior to giving birth and calculating their weight-change over their pregnancy. The second categorised women as falling within, below or above IOM guidelines, calculating the rate of weight change over the course of the pregnancy, and comparing this with the ranges provided by the IOM for each pre-pregnancy BMI category.

#### 2.2.4. Health-Related Behavioural Measures

Self-reported physical activity was assessed using questions about the number of 30-min sessions/week for walking and moderate physical activity and of 20-min sessions/week for vigorous physical activity. Self-reported dietary behaviour was assessed using questions from the NSW Population Health Survey, which measure daily servings of fruit and vegetables, weekly takeaway meals and daily soft drink consumption [23]. Adherence to the Australian guidelines for physical activity (150 min/week) [24] and fruit (2 servings per day) and vegetable consumption (5 servings per day) [25] was determined by calculating the total number of minutes of physical activity/week across any combination of walking, moderate and vigorous physical activity, and fruit and vegetable consumption by counts of serves/day. Average within-individual change for behavioural measures was calculated at baseline and either goal attainment or graduation.

### 2.3. Data Analysis

Descriptive statistics for the demographic profile of GHiP participants excluded missing data in keeping with other Get Healthy program evaluations [26]. Inferential analyses were conducted for engagement with the program and weight-related outcomes. Results for descriptive analyses were presented using counts and proportions for categorical data and means and standard deviations for continuous data. Logistic regressions were conducted to determine the association of dichotomously coded outcomes (program completion/not completing the program, meeting weight-gain guidelines/not meeting guidelines) with demographic characteristics. McNemar’s tests were conducted to assess within-individual change for health-related behavioural factors. For the tri-level GWG outcome (below, within and above guidelines), multinomial logistic regression was used (again with the same predictive demographic factors), with individuals whose weight-change fell within guidelines forming the reference category.

## 3. Results

### 3.1. Socio Demographic Characteristics of Participants

Of the 3702 women enrolled in the GHiP program throughout the evaluation period, more enrolled during 2019 (63.8%) than 2018 (36.1%, Table 1). On average, women who enrolled were 31.3 years old and were 22.6 weeks pregnant, and for over half (54.3%) it was their first pregnancy. The majority had a tertiary or vocational education (78.4%), were in paid employment (68.6%), spoke English at home (73.9%) and were from major cities (82.9%).

### 3.2. Participant Engagement and Program Completion

Almost all women who enrolled completed at least one coaching call (n = 3682, 99.5%). Of these, 61.5% (n = 2263) completed one to three calls, 25.6% completed four to seven calls (n = 944), and 12.9% completed eight to ten calls (n = 475). In the univariate analyses, women were significantly more likely to complete the GHIP program if they had a healthy pre-pregnancy weight, were older, had a certificate or tertiary level education, were located in major cities and were from the least disadvantaged areas. When the analysis was adjusted for all variables, only age and pre-pregnancy weight remained significant (Table 2). With every year of increasing age, women were more likely to complete the program (OR 1.05). Women with pre-pregnancy overweight (OR 0.77) or obesity (OR 0.66) were less likely to complete the program.

### 3.3. Health-Related Behavioural Outcomes Associated with the Get Healthy in Pregnancy Program

After participating in the GHiP program, significant improvements were noted in relation to the proportion of women meeting the guidelines for physical activity (from 39.7% to 50.5%); consuming the recommended servings of vegetables (from 13.2% to 30.8%); and consuming the recommended levels of fruit (from 67.4% to 74.4%) (all *p*-value < 0.001). Women who completed 10 calls made significant changes in higher-risk behaviours than those who attained their goal before completing 10 calls (Table 3). Those who reached their goal prior to the tenth coaching call reported on average, a statistically significant increase in vegetable consumption of 0.8 servings/day (*p* < 0.001) and a decrease in takeaway meals (0.4 meals/week, *p* < 0.001) and sweetened drinks (0.1 drink/day, *p* < 0.001, Table 3). Women who graduated also reported positive health-related behaviour changes in increased sessions of walking (0.8 session/week, *p* < 0.001), vigorous physical activity (0.3 sessions/week, *p* < 0.001), vegetable consumption (1.1 serving/day, *p* < 0.001), and decreased takeaway meals (0.8 meals/week, *p* < 0.001) and sweetened drinks (0.2 drinks/day, *p* < 0.001). Fruit consumption decreased (0.2 servings/day, *p* = 0.004).

### 3.4. Weight-Related Outcomes Associated with the Get Healthy in Pregnancy Program

Valid weight change data (participants who had recorded weight change prior to giving birth) was available for 245 women. Of these, 31% (n = 77) gained weight below GWG guidelines, 33% (n = 80) gained weight within GWG guidelines, and 36% (n = 88) gained weight above GWG guidelines (Table 4). Among women who gained weight within the guidelines, 61.3% (n = 49) had a pre-pregnancy weight within the healthy range, 17.5% (n = 14) had pre-pregnancy overweight and 13.7% (n = 11) had pre-pregnancy obesity. A larger proportion of women with GWG above the guidelines had pre-pregnancy overweight or obesity (60.3%, n = 53) than the proportion with a healthy pre-pregnancy BMI (38.6%, n = 38). For those with GWG below the guidelines, 40.3% (n = 31) had a healthy pre-pregnancy BMI, and 54.6% (n = 42) had pre-pregnancy overweight or obesity. 

Pre-pregnancy BMI was the only factor significantly associated with meeting weight-gain guidelines in the multivariable model. Women with pre-pregnancy obesity had approximately 70% lower odds of having weight gain within GWG guidelines, and those with overweight prior to pregnancy had approximately 60% lower odds of having a weight change within GWG guidelines compared with women who had a healthy pre-pregnancy BMI (Table 5). 

## 4. Discussion

This study reports on the impact of a population-wide health coaching program provided to pregnant women on individual level behaviour changes. Specifically, it reports on participant socio-demographic characteristics, levels of program engagement, and health-related behavioural and weight-related outcomes. Our analysis demonstrates the effectiveness of the program when delivered at scale for lifestyle-related behaviour changes, which aligns with findings of the GHiP pilot trial [17].

### 4.1. Program Effectiveness 

Our study found that there were positive shifts among GHiP program participants, both for those who graduated and those who achieved their nominated goals, in meeting the guidelines for physical activity, consuming the recommended servings of vegetables and decreasing takeaway meals and sweetened drinks overall. This impact may have been significant enough to make meaningful changes in weight-related outcomes. The majority of women did not gain weight above the guidelines, and our study showed that the GHiP program was effective in supporting GWG within the guidelines for approximately one third of women with pre-pregnancy overweight and obesity, however the dataset available was small. There was also a higher proportion of women with pre-pregnancy obesity who gained weight below the guidelines (33.8%) than above the guidelines (28.5%). Impacts of GWG below the IOM guidelines have not been conclusively associated with adverse pregnancy outcomes and as such, these must be further explored at both the individual and population level [27].

Although women who graduated demonstrated more changes in behavioural risk factors than those who left the program early, a dose–response relationship between behavioural outcomes and the number of coaching calls received was not evident. This may be due to the substantial variability in the stage of pregnancy at which women started (and finished) their involvement in the program. Qualitative research to explore the barriers and facilitators for program participation with both participants and health coaches could inform program improvements to enhance the effectiveness of GHiP as has been conducted by other lifestyle programs addressing GWG [28,29,30]. 

### 4.2. Program Engagement

Our study shows that most women enrolled in GHIP (60.4%) completed only one to three coaching calls. While this finding is common in real-world health promotion interventions, it requires further exploration. Women with pre-pregnancy obesity were less likely to complete four to seven (27.4%) and eight to ten (25.2%) coaching calls than those with a healthy pre-pregnancy weight (41.2% and 45.9%, respectively). Our analysis of a small number of women found no association between the number of coaching calls and women having GWG within the IOM guidelines. 

Systematic review evidence for the optimal dose of health coaching for obesity and type 2 diabetes interventions has identified that on average, 12–15 sessions of 35–40 min across 7–9 months are used in practice [31]. Moreover, a recent systematic review has identified six or more sessions as optimal in efficacious antenatal lifestyle interventions [32]. Given the emerging best practice guidelines for the dose of lifestyle interventions during pregnancy, further investigation into the optimal number of calls and gestational age for enrolment in the GHiP program is warranted. 

Evidence suggests that increasing fatigue and medical conditions are barriers for physical activity for pregnant women [28]. It is also important to consider the impact and complications of pregnancy generally with programs needing to be functional and flexible enough to tailor the schedule and intensity of intervention to a woman’s circumstances [28,33,34]. One Australian study that included an embedded prevention service in antenatal care found that the access to medical information and subsequent tailored coaching advice driven by a client’s clinical needs were enabling factors to maintaining healthy behaviour changes and satisfaction with the program [28].

Positive health-related behaviour changes were however reported by women who attained their goal early and who graduated (completed all calls). Women who graduated made statistically significant changes in more behavioural risk factors (walking, vigorous physical activity, vegetable consumption, takeaway meals and sweetened drink), than those who reached their goal after four calls but prior to ten calls (e.g., vegetable consumption, takeaway meals and sweetened drink). The literature identifies GWG as complex, and influenced by a number of factors, including demographic, physical, psychological and socio-cognitive factors, which have not been well included in the design of interventions to manage GWG [10]. As such, the findings related to program engagement and program completion warrant in-depth qualitative research with those women who are referred but do not engage, who are enrolled but do not complete the program, and who withdraw from the program. 

Our findings suggest that the reach of GHIP to date is not representative of the target population and may be lower than expected from a population-wide service. The majority of GHiP participants were from major cities, had a tertiary or vocational education, were in paid employment and spoke English at home. Similarly, the program is currently not well accessed by Aboriginal women who comprised less than 1% of the GHiP participants (in NSW between 2016 and 2020, 4.9% of all mothers giving birth were Aboriginal [35]. Other healthy lifestyle programs for pregnant women have also found that disadvantaged women who need support are most difficult to reach [36,37]. The literature identifies that Australian women from rural and remote areas, and areas of socioeconomic disadvantage have higher rates of being overweight and obese during pregnancy, highlighting the need to reach these population groups [38,39].

The findings, combined with recent research that lifestyle interventions during pregnancy implemented population-wide provide governments with cost savings and a good return on investment [40], and maternal and infant outcomes [6,13], support the continued provision of the GHiP program. However, the results of this evaluation indicate that increasing the reach of the program, as well as maintaining strong program referral and support for completing the program will be necessary for the program to have population-wide impact.

### 4.3. Limitations

This evaluation analyses data from the GHiP program, as implemented in a real-world setting, at scale. The data available in this analysis was collected using methods suitable for a telephone-based program. Identified limitations include the use of self-reported measures for weight, physical activity and dietary outcomes. While self-reported weight is considered reliable to classify BMI categories, women with pre-pregnancy overweight or obesity tend to underreport their pre-pregnancy weight [41]. The Australian guidelines for physical activity for pregnant women are relevant to those without contraindications [42], and as our analysis was not able to identify women with contraindications, we could not account for women who did not meet the guidelines for this reason. As participant data are deidentified, we are unable to link the participant data to their pregnancy outcomes and analyse the impact of the service on these. This is an area for future research that can potentially demonstrate the increased value of the service to women and antenatal care providers.

## 5. Conclusions

Participants in this evidence-based and scaled-up health coaching program demonstrated improved physical activity and dietary behaviours and the majority of participants did not gain weight above the guidelines. While based on a small sample size, the GHiP program has the potential to support pregnant women, including those with pre-pregnancy overweight and obesity in achieving GWG within IOM guidelines and improved lifestyle related behaviours to support a healthier pregnancy, which is known to have positive outcomes for both them and their baby. Key opportunities include increasing the population-level reach of the GHiP program, improving reach to broader priority populations and exploring ways to increase engagement for women who could most benefit from the program.

## Figures and Tables

**Table 1:** Characteristics of Get Healthy in Pregnancy program participants ^1^.

Characteristics		Mean	SD
Age (n = 3641)	Min 16.5 years	31.3	5.1
	Max 48.9 years		
Gestational age (n = 3529)	Min 5 weeks	22.6	6.2
	Max 40 weeks		
		**n**	**%**
Year of enrolment (n = 3702)	2018	1339	
	2019	2363	
First pregnancy (n = 3653)	Yes	1982	54.3
	No	1671	45.7
Highest education (n = 3466)	High school	748	21.6
	Tertiary/vocational	2718	78.4
Employment (n = 3546)	Paid employment	2434	68.6
	No paid employment	1112	31.4
Language spoken at home (n = 3604)	English	2663	73.9
	Other	941	26.1
Aboriginal status ^2^ (n = 3610)	Aboriginal	25	0.7
	Non-Aboriginal	3585	99.3
ARIA ^3^ (n = 3610)	Major Cities	2994	82.9
	Inner regional	508	14.1
	Outer regional	104	2.9
	Remote/very remote	4	0.1
SEIFA IRSD ^4^ (n = 3673)	1-quintile ^most disadvantaged^	682	18.6
	2-quintile	703	19.1
	3-quintile	766	20.9
	4-quintile	477	13.0
	5-quintile ^least disadvantaged^	1045	28.5

^1^ This table includes all participants who enrolled from 1 January 2018 to 31 December 2019. ^2^ Aboriginal and Torres Strait Islander people are referred to as Aboriginal people in recognition that Aboriginal people are the original inhabitants of NSW. ^3^ ARIA is a measure of geographical remoteness. ^4^ SEIFA IRSD (Socio-Economic Indexes, Index of Relative Socio-economic Disadvantage for Areas) provides a summary of people living in an area representing the general level of socio-economic disadvantage of all people in that area.

**Table 2:** Multivariable model estimates of associations between participant characteristics and program completion.

	Multivariable Logistic Regression		
Variable	Odds Ratio (95% CI)	z-Value	*p*-Value
			
**Age (years)**	1.045 (1.03–1.06)	4.81	<0.001
**Weeks pregnant at baseline**	0.994 (0.98–1.01)	−0.86	0.39
**BMI Category pre-pregnancy (ref: healthy weight)**			
Underweight	0.884 (0.58–1.35)	−0.57	0.57
Overweight	0.767 (0.62–0.95)	−2.49	0.01
Obese	0.662 (0.53–0.82)	−3.73	<0.001
**Highest level of education completed (ref: ≤Year 12)**			
Tertiary education or certificate	1.203 (0.95–1.53)	1.52	0.13
**Employment (ref: paid employment)**			
No paid employment	1.201 (0.99–1.46)	1.88	0.06
**ARIA ^1^ category (ref: major cities)**			
Other regions	0.961 (0.75–1.24)	−0.31	0.76
**SEIFA ^2^ (ref: most disadvantaged 40%)**			
Least disadvantaged 60%	1.028 (0.85–1.24)	0.29	0.77
**Language (ref: English)**			
Language other than English	1.237 (1.02–1.51)	2.12	0.03

^1^ ARIA is a measure of geographical remoteness. ‘Other’ comprised inner regional, outer regional, rural and remote categories. ^2^ SEIFA IRSD (Socio-Economic Indexes, Index of Relative Socio-economic Disadvantage for Areas) provides a summary of people living in an area representing the general level of socio-economic disadvantage of all people in that area.

**Table 3:** Within individual change for behavioural risk factors from baseline to early goal attainment, and baseline to graduation.

Behavioural Risk Factors	Total Matched Pairs n = 363						Total Matched Pairs n = 293					
		Baseline		Early Goal Attainment				Baseline		Graduation		
	n	Mean	SD	Mean	SD	*p*-Value	n	Mean	SD	Mean	SD	*p*-Value
Walking (number of 30 min sessions/week)	363	2.9	2.6	3.2	2.7	0.02	293	2.8	2.8	3.6	2.7	<0.001
Moderate PA (number of 30 min sessions/week)	362	0.8	1.6	0.9	1.7	0.08	293	0.7	1.6	1.0	2.0	0.003
Vigorous PA (number of 20 min sessions/week)	358	0.1	0.7	0.1	0.6	0.84	288	0.0	0.4	0.3	1.1	<0.001
Vegetable consumption (number of servings/day)	357	2.9	1.6	3.7	1.6	<0.001	280	2.7	1.5	3.8	1.6	<0.001
Fruit consumption (number of servings/day)	358	2.0	1.2	2.1	1.0	0.27	357	2.1	1.2	1.9	1.0	0.004
Takeaway meals (number of meals/week)	356	1.4	1.7	1.0	1.4	<0.001	280	1.6	3.7	0.8	1.3	<0 001
Sweetened drinks (number of drinks/day)	355	0.3	0.8	0.2	0.6	<0.001	280	0.4	0.9	0.2	0.7	<0.001

Note: This table includes participants who provided physical activity, and fruit and vegetable consumption data. PA = physical activity. Early goal attainment = reached goal prior to completing 10 coaching calls. Graduation = completed 10 calls.

**Table 4:** Pre-pregnancy BMI of participants by weight change during pregnancy.

Weight Change during Pregnancy	Below Guidelines		Within Guidelines		Above Guidelines	
	Mean	SD	Mean	SD	Mean	SD
Age in years	31.8	4.9	32.7	4.5	31.5	5.6
Weeks pregnant at baseline	21.9	4.8	21.0	4.2	20.8	4.2
	**n**	**%**	**n**	**%**	**n**	**%**
BMI category pre-pregnancy						
Underweight	4	5.2	6	7.5	1	1.1
Healthy	31	40.3	49	61.3	34	38.6
Overweight	16	20.8	14	17.5	28	31.8
Obese	26	33.8	11	13.7	25	28.5
Total n = 245	77		80		88	

**Table 5:** Univariate and multivariable relationships between participant characteristics and weight outcomes.

	Univariate Models			Multivariable Model		
Variable	Odds Ratio (95% CI)	z-Value	*p*-Value	Odds Ratio (95% CI)	z-Value	*p*-Value
**Age**	1.043 (0.99–1.10)	1.53	0.13	1.016 (0.96–1.08)	0.49	0.62
**Number of coaching calls completed**	1.076 (0.94–1.24)	1.02	0.31	1.063 (0.91–1.24)	0.79	0.43
**Weeks pregnant at baseline**	0.989 (0.93–1.05)	−0.34	0.73	0.987 (0.92–1.06)	−0.37	0.71
**Pre-pregnancy BMI (ref: Healthy weight)**						
Underweight	1.650 (0.48–5.72)	0.79	0.43	1.666 (0.46–6.07)	0.77	0.44
Overweight	0.438 (0.22–0.89)	−2.29	0.02	0.414 (0.20–0.87)	−2.34	0.02
Obese	0.297 (0.14–0.63)	−3.18	0.001	0.305 (0.14–0.67)	−2.96	0.003
**Highest education (ref: Year 12 or lower)**						
Tertiary or certificate	2.269 (0.95–5.42)	1.84	0.07	1.818 (0.70–4.70)	1.23	0.22
**Employment (ref: Paid employment)**						
No paid employment	1.109 (0.63–1.94)	0.36	0.72	1.607 (0.84–3.07)	1.44	0.15
**SEIFA ^1^ (ref: Most disadvantaged 40%)**						
Least disadvantaged 60%	1.516 (0.83–2.76)	1.36	0.18	1.237 (0.64–2.41)	0.63	0.53
**ARIA ^2^ (ref: Major cities)**						
Other	0.580 (0.25–1.34)	−1.27	0.20	0.700 (0.28–1.76)	−0.76	0.45
**Language (ref: English speaking)**						
Language other than English	0.893 (0.50–1.60)	−0.38	0.70	0.610 (0.31–1.20)	−1.44	0.15

Note: Results indicate the odds of having weight gain within GWG guidelines. Values < 1 indicate a reduction, odds > 1 indicate increased likelihood relative to the reference category (for categorical variables) or for a 1-unit increase (continuous variables). ^1^ SEIFA IRSD (Socio-Economic Indexes, Index of Relative Socio-economic Disadvantage for Areas) provides a summary of people living in an area representing the general level of socio-economic disadvantage of all people in that area. ^2^ ARIA is a measure of geographical remoteness. ‘Other’ is comprised of inner regional, outer regional, rural and remote categories.

## Data Availability

The data presented in this study are not publicly available as participants whose data were included in the analysis have not consented to data sharing.
